# Mentalization in developmental age’s eating disorders: Comparison between anorexia nervosa and avoidant/restrictive food intake disorder (ARFID)

**DOI:** 10.1192/j.eurpsy.2021.252

**Published:** 2021-08-13

**Authors:** F. Gigliotti, C. Basile, M. Colaiori, A. Terrinoni, I. Ardizzone, F. Di Santo

**Affiliations:** Department Of Human Neuroscience, Section Of Child And Adolescent Neuropsychiatry, Sapienza University of Rome, Rome, Italy

**Keywords:** eating disorders, mentalization, anorexia nervosa, ARFID

## Abstract

**Introduction:**

Anorexia Nervosa (AN) and Avoidant/Restrictive Food Intake Disorder (ARFID) are two primary restrictive eating disorders described in DSM-5, characterized both of them by insufficient food intake. This behavior In ARFID is not driven by weight and shape concerns that tipify AN. While there are several studies that highlight the presence of mentalizing difficulties in AN, there are still no data about mentalizing profile in ARFID.

**Objectives:**

The aim of this study was to better characterize the mentalizing profile of AN and ARFID children and adolescent.

**Methods:**

Two groups of AN or ARFID outpatients (15+15), aged 6 to 18 years, were assessed by Alexythimia Questionnaire for Children (AQC) and Toronto Alexythimia Scale-20 (TAS-20) to evaluate alexythimia; by Interpersonal Reactivity Index (IRI) and Basic Empathy Scale (BES) to assess empathy; by NEPSY-II social perception subtests to evaluate Theory of Mind and Emotion recognition. Exclusion criteria were the presence of intellectual disability, pervasive developmental disorders and binge eating behavior (eating disorder other than AN or ARFID).

**Results:**

Preliminary results showed different mentalizing profiles between ARFID and AN patients, with differences in the score for affective empathy, lower in ARFID than in AN patients while the score for alexythimia traits resulted higher in AN population.

**Conclusions:**

By our results, mentalization impairment appeared trans-diagnostic across several eating disorders. This first result should be further improved to better analyze this construct in order to develop effective clinical intervention to improve the subject’s affective regulation.

**Disclosure:**

No significant relationships.

